# Dynamic Changes in Rumen Microbial Diversity and Community Composition Within Rumen Fluid in Response to Various Storage Temperatures and Preservation Times

**DOI:** 10.3390/vetsci12030234

**Published:** 2025-03-03

**Authors:** Chang Liu, Jin Cheng, Yunong Xie, Kehui Ouyang, Mingren Qu, Ke Pan, Qinghua Qiu

**Affiliations:** Jiangxi Province Key Laboratory of Animal Nutrition and Feed, College of Animal Science and Technology, Jiangxi Agricultural University, Nanchang 330045, China

**Keywords:** bacterial community composition, bacterial diversity, preservation time, quorum sensing, rumen fluid, storage temperature

## Abstract

The aim of this study was to investigate the effects of storage temperature and preservation time on the microbial diversity and community composition of rumen fluid. Rumen fluid samples were collected from six Hu sheep fed on a high-forage diet and stored at −80 °C and −20 °C for intervals of 0, 7, 14, 30, 60, 120, and 240 days. DNA was extracted at each time point for 16S rRNA gene sequencing to evaluate the rumen microbial diversity and community composition. The results showed that storage temperature affected only the relative abundance of Proteobacteria, with no substantial impact on alpha-diversity or other microbial groups (*p* > 0.05), and no significant interaction effects were observed between storage temperature and preservation time (*p* > 0.05). Alpha-diversity indices such as Chao1, observed species, and PD whole tree showed dynamic changes after 7 days of storage, while the relative abundances of Verrucomicrobiota and *Christensenellaceae R-7 group*, as well as the energy metabolism metabolic pathway, exhibited significant alterations after 14 days of storage (*p* < 0.05). Notably, Patescibacteria, *Rikenellaceae RC9 gut group*, and *Veillonellaceae UCG-001* abundances demonstrated significant changes after 240 days of storage (*p* < 0.05). Both principal coordinates analysis (PCoA) and non-metric multidimensional scaling (NMDS) showed distinct overlaps. This study suggests that storing rumen fluid at −80 °C and −20 °C does not influence rumen microbial diversity and community composition, whereas the storage time significantly impacts these factors, with most differences emerging after 14 days of preservation. Consequently, it is advised that the analysis of microbial diversity and community composition in rumen fluid samples be conducted within 14 days post-collection.

## Simple Summary

In the realm of ruminant nutrition research, collecting rumen fluid samples at various time points is commonly undertaken. However, the need for immediate microbial analysis post-collection is questionable. This raises the question of whether the duration of sample storage affects the results of the microbiome analysis. Understanding how microbes in rumen fluid samples change over time is essential for resolving this question. Thus, the aim of this study is to monitor the changes in microbial diversity and community composition within rumen fluid as influenced by storage time and temperature, with the goal of providing scientific decisions regarding the optimal storage conditions for rumen fluid. Fresh rumen fluid samples were stored at −80 °C and −20 °C for periods of 0, 7, 14, 30, 60, 120, and 240 days. Our results showed that the storage temperature did not significantly affect microbial diversity or community composition, but there was a notable shift in the diversity of rumen microbes and certain species of bacteria after 14 days of storage. Therefore, it is recommended that rumen fluid collected for microbial analysis be stored at −80 °C or −20 °C and that the analysis be conducted within 14 days to maintain the accuracy of the results.

## 1. Introduction

Rumen microbial ecology represents a highly complex and dynamic system where diverse microbial taxa play essential roles in digestion and animal health [1]. The rumen is a rich habitat for a vast array of microorganisms, including bacteria, archaea, fungi, and protozoa [2]. Among these, several key taxa are particularly abundant and functionally significant. For example, *Prevotella* is a dominant genus, central to carbohydrate and hydrogen metabolism; *Ruminococcus* is renowned for its cellulolytic and amylolytic activities, which are vital for breaking down plant materials; and *Butyrivibrio* is crucial for degrading xylans, pectins, and hemicellulose, as well as for protein breakdown and biohydrogenation of fatty acids [3]. Methanogenic archaea, such as *Methanobacterium* and *Methanothermobacter*, are also highly prevalent, removing hydrogen to support fermentation and methane production [4]. These microbial taxa interact in intricate ways, enhancing nutrient utilization and promoting overall host health.

Rumen fluid is pivotal in nutritional research concerning ruminants and serves as the epicenter of microbial fermentation, which is essential for their digestive processes [5]. Within this complex ecosystem, the breakdown of ingested plant material takes place, resulting in the production of critical nutrients, including volatile fatty acids and amino acids, that are vital for ruminant energy and protein metabolism [6]. The composition and activity of the rumen microbiota are significantly influenced by factors such as environmental factors, including climate, diet, and agricultural practices [7]. The microbiome in rumen fluid is both diverse and highly adaptive, reflecting the host animal’s dietary habits and responses to environmental conditions [8]. Therefore, a deep understanding of these interactions is crucial for improving ruminant nutrition and enhancing production efficiency.

In the field of animal nutrition research, the high cost of sequencing often requires waiting for results from other indicators—such as growth performance, meat quality, and rumen fermentation characteristics—before deciding to proceed with rumen microbiome sequencing. Additionally, studies that necessitate the collection of rumen fluid samples at various times encounter the dilemma of whether to send the samples for immediate analysis or to freeze them for batch processing at the trial’s end. Resolving these challenges hinges on one critical question: whether the diversity and composition of rumen microbes significantly change during storage. In fact, some studies have already explored the changes in some microbes in the rumen fluid during storage. A previous study found that the total viable bacterial count and colony-forming units were similar when rumen fluid was stored at 0 °C for 8 h [9]. Martin et al. [10] investigated the microbiological stability of rumen fluid over a 24-h period at temperatures ranging from −18 °C to 38 °C and determined that storage at 38 °C for up to 9 h or 2 h at room temperature maintained viabilities comparable to those of fresh rumen fluid. Takizawa et al. [11] reported that rumen fluid kept at 4 °C for a week retained significant fibrolytic activity and served as a suitable organic carbon source for methane fermentation of wastepaper. However, another study also found a reduction in microbial activity when fresh rumen fluid underwent freezing or lyophilization [12]. Tunkala et al. [13] reported that the protozoa population in rumen fluid decreased sharply after being preserved at −80 °C or −20 °C for 4 and 8 days, and dynamic changes in in vitro fermentation parameters were observed when rumen fluid was preserved at −20 °C for different days [14]. Spanghero et al. [15] found that the relative abundance of *Fibrobacter succinogenes* decreased when rumen fluid was chilled at −80 °C compared to when it was refrigerated at 4 °C. Additionally, preserving rumen fluid at room temperature or at −80 °C did not significantly affect the sample clustering and the quantification of Firmicutes and Bacteroidetes [16].

However, the aforementioned studies only focused on changes in specific microbes without exploring the microbial community as a whole. In our previous study on the effects of storage time on rumen fluid under a high-concentrate diet, we observed a trend where microbial richness varied with storage duration [17]. It was reported that the protozoa population stabilized after being stored at −20 °C or −80 °C for 8 days in rumen fluid collected from cows fed a high-forage diet [13]. Other research has also indicated that the physicochemical properties of rumen fluid change over time in relation to diet type [10]. Given the substantial impact of diet on rumen microbial diversity and community composition, its response to storage time may also be diet-dependent. Consequently, it is still essential to investigate the dynamic changes in rumen fluid over time under high-forage feeding regimens.

Samples are well-suited for long-term preservation at −80 °C [18], but this option comes with drawbacks such as high maintenance costs and limited storage capacity. In contrast, −20 °C freezers, which are commonly available in laboratories, offer more spacious storage and are more cost-effective to maintain. This contrast leads us to investigate whether these two distinct freezing conditions have any influence on the microbial community of rumen fluid and whether there is a significant interaction with the duration of storage time. The aim of this study is to elucidate how microbial diversity and community composition in rumen fluid, especially under high-forage feeding regimens, are affected by storage temperature and time, thereby providing guidance on the optimal timing for rumen fluid analysis. It is hypothesized that while the storage temperatures of −80 °C and −20 °C may not independently affect the microbes, the diversity and community composition of the rumen microbiota are likely to change significantly over the course of storage time.

## 2. Materials and Methods

### 2.1. Animal Ethics

The Committee for the Care and Use of Experimental Animals at Jiangxi Agricultural University provided the animal care and welfare guidelines in accordance with Protocol Number JXAULL-2021036 approved on 5 March 2021.

### 2.2. Rumen Fluid Acquisition

Rumen fluid was collected from six sheep (body weight 30.65 ± 1.30 kg) fed a high-forage diet with a roughage/concentrate ratio of 69:31 for three months. The dietary composition was meticulously crafted from the following ingredients in their respective proportions: peanut straw, which constituted the majority at 68.58%, was accompanied by corn at 11.22%, soybean meal at 11.17%, wheat bran at 4.65%, calcium hydrogen phosphate at 0.15%, sodium bicarbonate at 0.24%, salt at 0.49%, and a premix at 3.50%. The peanut straw was composed of stems and leaves, and its chemical composition included the following: 112.2 g/kg of crude protein, 114.7 g/kg of crude ash, 480.7 g/kg of neutral detergent fiber, and 324.8 g/kg of acid detergent fiber. This blend provided a balanced array of nutrients, including a metabolizable energy content of 8.08 MJ/kg, crude protein at 146.4 g/kg, neutral detergent fiber at 406.7 g/kg, and acid detergent fiber at 298.7 g/kg, ensuring a wholesome and nutritious diet for the sheep. The rumen contents were meticulously harvested at the moment of slaughter, ensuring a representative sample by gathering equal portions from the dorsal, central, and ventral sections. This mixed collection was then comprehensively blended to achieve a homogeneous mixture, and it was then filtered through a quadruple layer of cheesecloth to obtain the rumen fluid. This freshly extracted rumen fluid was categorized as the initial time point D0. To preserve the integrity of the samples, the rumen fluid from each sheep was portioned into several cryovials and quickly stored in ultra-low temperature storage at −80 °C and −20 °C, safeguarding the samples for future analysis.

### 2.3. Experimental Design

The rumen fluid samples were placed in a compartmentalized freezing box simultaneously to reduce the impact of temperature inhomogeneity in different spaces of the freezer on the freezing effect. Additionally, the samples from each individual were evenly distributed within the freezing box to further minimize the impact of temperature differences within the box on the freezing effect. The freshly collected rumen fluid was categorized as D0. Acting as a standard for preservation, the rumen fluid kept at −80 °C was identified with an initial ‘R’. This fluid underwent various storage times: 7 days (D7), 14 days (D14), 30 days (D30), 60 days (D60), 120 days (D120), and 240 days (D240). Each storage time point was assigned a unique label, creating a distinct set of codes for the −80 °C storage series: RD7, RD14, RD30, RD60, RD120, and RD240. In contrast, the rumen fluid preserved at −20 °C, representing a higher temperature condition, is marked with the prefix ‘H’. These samples were assigned labels that correspond to the duration-based nomenclature of the −20 °C group: HD7, HD14, HD30, HD60, HD120, and HD240, each matching the respective storage times of their −20 °C counterparts.

### 2.4. DNA Extraction, Sequencing, and Data Analysis

A total of 54 rumen fluid samples were employed for DNA extraction. These samples were sourced from each storage time point across both temperatures. For the first five time points, there were six samples each, which were combined into three samples per temperature according to a specific protocol: Samples 1 and 6 were combined to create a new Sample 1, Samples 2 and 5 to create a new Sample 2, and Samples 3 and 4 to create a new Sample 3. For the D120 and D240 time points, there were 12 samples each, with 6 rumen fluid samples per temperature. DNA extraction was conducted using a bacterial DNA Kit (OMEGA, Omega Bio-Tek, Norcross, GA, USA), and the procedure was carried out strictly in accordance with the instructions provided in the kit. The DNA quality and concentration were assessed using a Nanodrop 2000 spectrophotometer (ThermoFisher Scientific, Inc., Waltham, MA, USA).

The amplification of the V3-V4 region of the bacterial 16S rRNA gene was achieved using universal primers 338F (5′-ACTCCTACGGGAGGCAGCAG-3′) and 806R (5′-GGACTACHVGGGTWTCTAAT-3′). To distinguish between samples, an 8-base pair (bp) barcode sequence was added to the 5′ end of the forward and reverse primers. These primers, now with barcode sequences, were synthesized and amplified using an ABI 9700 PCR instrument (Applied Biosystems, Inc., Foster City, CA, USA), following the reaction conditions and protocols specified by Wei et al. [19]. The sizes of the amplified target bands were confirmed by 1% agarose gel electrophoresis, and the PCR products were automatically purified using the Agencourt AMPure XP DNA purification kit (Beckman Coulter, Inc., Brea, CA, USA). Library construction from the PCR products was carried out with an NEB Next Ultra II DNA Library Prep kit (New England Biolabs, Inc., Ipswich, MA, USA), and the constructed libraries were further purified using the Agencourt AMPure XP kit. The library fragment sizes were evaluated using an Agilent 2100 Bioanalyzer (Agilent Technologies, Inc., Santa Clara, CA, USA), and the library concentration was quantified using the ABI StepOnePlus Real-Time PCR System (Applied Biosystems, Inc., Foster City, CA, USA). The libraries were sequenced on the Nextseq 2000 platform (Illumina, Inc., San Diego, CA, USA) using a paired-end 300-base pair (PE300) strategy. The raw sequencing data have been deposited in the NCBI’s Sequence Read Archive (SRA) database under accession number PRJNA982413.

The initial phase of data processing involved sorting the raw sequencing data according to the barcode sequences, which correspond to individual samples. Subsequently, the PEAR software (version 0.9.11) was used to meticulously filter and assemble the sequences. This process included the removal of sequences containing the ambiguous base ‘N’ and the trimming of segments with quality scores below 20. The assembly criteria were stringent, requiring a minimum overlap of 10 base pairs and allowing only a 0.10 mismatch rate. Following assembly, Vsearch software (version 2.15.1) was used to remove sequences that did not meet the 260 bp threshold and to rigorously identify and excise chimeric sequences. This was achieved through precise alignment with the Gold Database, using the UCHIME method for accuracy. To further refine the data, the Usearch software (version 10.0.240) unoise3 algorithm was applied to the assembled sequences, reducing noise and resulting in the derivation of amplicon sequence variants (ASVs). These ASVs were then aligned with the SILVA 138 database using BLAST (version 2.15.0), a critical step for assigning taxonomic classifications to each variant. The taxonomic information derived from the ASVs was utilized with QIIME2 (version 2020.11) to calculate alpha diversity indices and construct beta diversity distance matrices. R software (version 4.1.2) was then employed to conduct a principal coordinate analysis (PCoA) based on the Bray–Curtis distance matrix, providing a visual representation of the data. To further explore group dynamics, non-metric multidimensional scaling (NMDS) was performed, with the vegan and ggplot2 packages in R facilitating both analysis and visualization. The analysis of similarities (ANOSIM) was also conducted to assess the degree of similarity between different groups, using the vegan package in R for analysis. In pursuit of a comprehensive examination of species-level differences across various taxonomic ranks within the treatment groups, the LEfSe analysis was conducted using Python (version 3.10.2). This analysis focused on identifying species with an LDA score above the threshold of 3.0, indicating a significant difference in abundance between groups.

### 2.5. Statistical Analyses

Data analysis was conducted using the MIXED Models procedure in SPSS (version 21, IBM Corporation, Armonk, NY, USA). The statistical model is formulated as follows: Y*_ijt_* = *μ* + T*_i_* + D*_t_* + A*_j_* + (TD)*_it_* + e*_ijt_*. In this model, Y*_ijt_* represents the dependent variables, μ is the overall mean, T*_i_* is the fixed effect of the preservation temperature, D*_t_* is the fixed effect of the preservation time, A*_j_* is the random effect of the animal, (TD)*_it_* is the interaction effect between preservation temperature and storage time, and e*_ijt_* is the random error. Furthermore, to distinctly identify the variations in both diversity and composition of the rumen microbiota between frozen rumen fluid and fresh rumen fluid, a series of pairwise comparisons were conducted. These analyses were based on the contrast between the frozen storage time points and the fresh rumen microbiota at the initial point D0, with the resulting differences indicated by P0. Statistical significance was defined as a *p*-value threshold of less than 0.05. Given that our study includes only two storage temperatures, Tukey’s multiple comparison test was selectively applied to those indicators showing significant differences in storage time. These significant findings are highlighted with the use of different lowercase letter annotations.

## 3. Results

### 3.1. Rumen Bacterial Alpha-Diversity

As shown in Table 1, the storage temperature of rumen fluid had no significant effect on the richness and evenness of rumen microorganisms, nor did it have an interaction with the storage time (*p* > 0.05). However, the storage temperature did have a significant impact on the Chao1, observed species, and phylogenetic diversity (PD) whole tree indices (*p* < 0.05). Specifically, the values for Chao1 and observed species at D14 and D120 were significantly higher than those at D7 and D60. Additionally, the PD whole tree index at D120 was significantly higher than that at D60.

### 3.2. Rumen Bacterial Community Composition

The effects of rumen fluid storage time and temperature on the rumen bacterial community composition at the phylum and genus levels are listed in Table 2 and Table 3, respectively. At the phylum level, with the exception of the Proteobacteria phylum, the storage temperature did not significantly impact the relative abundance of phyla with more than 0.1% relative abundance (*p* > 0.05). Additionally, no interaction effects between storage temperature and time were observed for these phyla (*p* > 0.05). The relative abundance of Proteobacteria was higher when the rumen fluid was stored at −80 °C compared to −20 °C (*p* < 0.05). Storage time significantly affected Proteobacteria, Verrucomicrobiota, and Patescibacteria (*p* < 0.05). Specifically, the relative abundance of Proteobacteria at D0 was significantly higher than at D7. The relative abundance of Verrucomicrobiota at D0 and D120 was significantly higher than at D14. Additionally, the relative abundance of Patescibacteria at D240 was significantly higher than at D0, D7, D14, and D30. Similarly, the storage temperature had no significant impact on the relative abundance of genera with more than 1.0% relative abundance, and no interaction effects between storage temperature and time were observed (*p* > 0.05). Storage time significantly affected the relative abundance of three genera: *Rikenellaceae RC9 gut group*, *Christensenellaceae R-7 group*, and *Veillonellaceae UCG-001* (*p* < 0.05). Specifically, the relative abundance of *Rikenellaceae RC9 gut group* and *Veillonellaceae UCG-001* at D240 was significantly higher than at D0, while the relative abundance of *Christensenellaceae R-7 group* at D0 was significantly higher than at D14 and D30.

### 3.3. Rumen Bacterial Beta-Diversity

**Figure 1:**
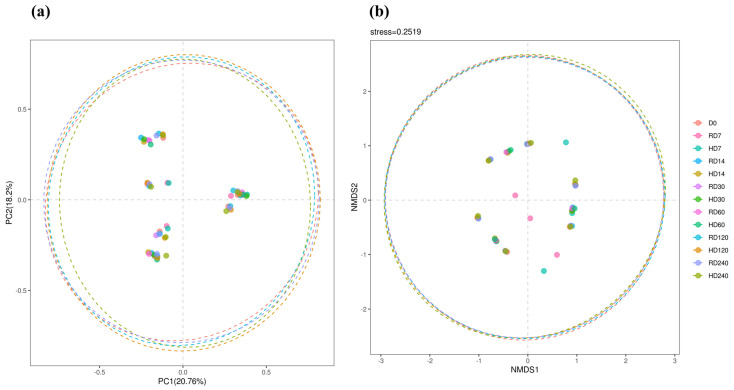
Principal coordinates analysis (PCoA, (**a**)) and non-metric multidimensional scaling (NMDS, (**b**)) of rumen bacterial community within rumen fluid from various preservation times and storage temperatures.

Figure 1 illustrates that both principal coordinate analysis (PCoA) and non-metric multidimensional scaling (NMDS) reveal a pronounced overlap across the groups. The analysis of similarities (ANOSIM) pairwise comparisons revealed significant differences at the −80 °C mark, particularly between D0 and D7 (R = 0.5802, *p* = 0.011), D7 and D120 (R = 0.5926, *p* = 0.010), and D7 and D120 at −20 °C (R = 0.5864, *p* = 0.012). Additionally, significant differences are observed between D7 at −80 °C and D240 at −80 °C (R = 0.5864, *p* = 0.014), as well as between D7 at −80 °C and D240 at −20 °C (R = 0.5988, *p* = 0.010).

### 3.4. Biomarker Microbes

**Figure 2:**
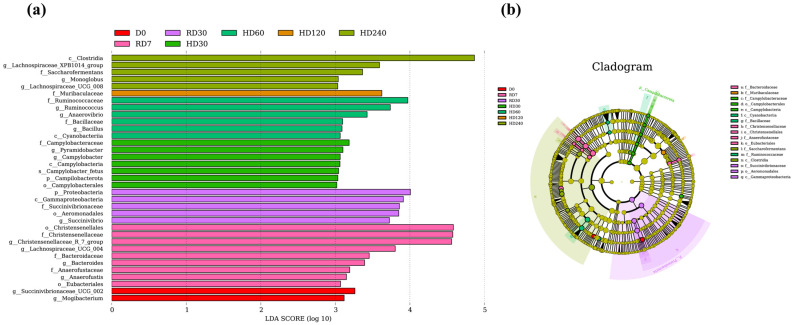
Effect of preservation time and storage temperature on the discriminative bacterial communities across various taxonomic levels in rumen fluid is illustrated by (**a**) linear discriminant analysis and (**b**) cladogram.

The LDA distribution bar chart and cladogram illustrate that a total of thirty-eight species were identified (Figure 2). In addition to the differential species and their associated taxonomic groups previously mentioned at the phylum and genus levels, thirty additional species have been identified as differential microbes. These differential microbes included g_*Succinivibrionaceae UCG-002* and g_*Mogibacterium* in the D0 group; g_*Lachnospiraceae UCG-004*, f_Bacteroidaceae, g_*Bacteroides*, f_Anaerofustaceae, g_*Anaerofustis*, and o_Eubacteriales in the RD7 group; c_Gammaproteobacteria, f_Succinivibrionaceae, o_Aeromonadales, and g_*Succinivibrio* in the RD30 group; f_Campylobacteraceae, g_*Pyramidobacter*, g_*Campylobacter*, s_*Campylobacter fetus*, p_Campilobacterota, and o_Campylobacterales in the HD30 group; f_Ruminococcaceae, g_*Ruminococcus*, g_*Anaerovibrio*, f_Bacillaceae, g_*Bacillus*, and c_Cyanobacteria in the HD60 group; f_Muribaculaceae in the HD120 group, and c_Clostridia, g_*Lachnospiraceae XPB1014 group*, f_Saccharofermentans, g_*Monoglobus*, and g_*Lachnospiraceae UCG-008* in the HD240 group.

### 3.5. Rumen Bacteria Predicted Metabolic Pathway

The effects of rumen fluid storage time and temperature on the relative abundance of the predicted metabolic pathways in the rumen bacterial microbiome are presented in Table 4. The storage temperature had no significant effect on the predicted metabolic pathways, and no interaction effect between storage time and temperature was observed (*p* > 0.05). The metabolic pathway of the energy metabolism in D0 was lower than that in D14, D60, and D120 (*p* < 0.05). The relative abundance of the translation pathway in D7 was higher than that in D14, D30, D120, and D240 (*p* < 0.05).

## 4. Discussion

Theoretically, the sizes of ice crystals formed at different temperatures vary. At higher sub-zero temperatures, such as −10 °C, slower cooling rates result in larger ice crystals. These larger ice crystals can cause mechanical damage to microbial cells, disrupting cell membranes and organelles [20]. In contrast, at lower temperatures like −80 °C, faster cooling leads to the formation of smaller ice crystals. This reduces mechanical stress on the cells and helps preserve cellular integrity [20]. These differences in ice crystal formation may affect microbial viability and metabolic activity, which in turn can influence microbial diversity and structures. The experiment was designed with two specific storage temperatures, −80 °C and −20 °C, to address the issue of limited sample storage space in laboratories. Since rumen fluid samples were preserved in a frozen state at both temperatures, microbial diversity and the relative abundance of the majority of microorganisms remained stable, possibly due to the similar size of ice crystals and the stability of DNA during freezing. This observation suggests that the −20 °C freezer can serve as a viable alternative for storing rumen fluid samples when −80 °C freezer space is limited. Chao1, observed species, and PD whole tree are widely used metrics for assessing microbial richness and ecosystem stability [21]. Elevated values of these indices suggest higher microbial richness or greater stability, while lower values indicate a reduction in microbial diversity or potential threats to the micro-ecosystem. In this study, these indices showed fluctuations over a 240-day storage period, initially declining, then rising, followed by another decline before stabilizing. The initial drop within the first seven days is likely due to the transition of rumen fluid from room temperature to a frozen state, which causes temperature stress, resulting in decreased diversity. Subsequently, microbes may employ community defense mechanisms, such as quorum sensing, to cope with adversity [22]. By day 60, the depletion of feed residue in the rumen fluid, along with nutrient scarcity, leads to a further decrease in microbial diversity [23]. However, after adapting collectively to low-nutrient conditions, diversity rebounds to its original level. This dynamic shift illustrates the adaptive responses of rumen microbes to temperature fluctuations and nutrient limitations, underscoring the complexity of their survival strategies.

Proteobacteria exhibit an adaptive capacity to environmental temperature fluctuations, typically showing a decreased relative abundance under higher temperatures [24]. In this study, it was the only microorganism identified as being influenced by the temperature treatments applied. Notably, its relative abundance was lower at −20 °C compared to −80 °C under storage conditions, which is consistent with previous research findings. Throughout the environmental adaptation process, certain strains of Proteobacteria that fail to adapt are phased out [25]. This study evidences this trend with a dip in relative abundance at D7 and D14, followed by a rebound to prior levels by D30 as the bacteria gradually acclimate, with microbial diversity also resuming to its initial state. Subsequently, as nutrients in the rumen fluid are exhausted, their abundance declines steadily. The dynamic changes in Proteobacteria over the storage period underscore the intricate ties between Proteobacteria, environmental pressures, nutritional availability, and the diversity of the microbial community. Verrucomicrobiota, known for its broad distribution across various ecosystems, specializes in the degradation of organic polymers and possesses the ability to break down polysaccharides [26]. During the first 14 days of rumen fluid storage, the organic matter and polysaccharides from residual feed are progressively consumed by microorganisms, resulting in a gradual decrease in the relative abundance of Verrucomicrobiota. Interestingly, from day 30 to day 120, there is an observed increase in their numbers, possibly due to the bacterium’s capacity to utilize volatile fatty acids produced by other microbes as nutrients for its own reproduction. This hypothesis is supported by previous findings that report an increase in the levels of volatile fatty acids during this period [23]. Patescibacteria is characterized by its distinct ribosomal RNA (rRNA) genes and ribosome architecture, as well as its limited metabolic capacity, compact genome, and small cell size [27]. Genomically, it shows a reduction in functions related to motility and flagella, outer membrane components, polysaccharide metabolism, biosynthesis pathways, and nutrient transport systems [27], indicating that this bacterium might depend on hosts or other microorganisms for nutrient acquisition in cold environments. Throughout the storage of rumen fluid, Patescibacteria appear to acclimatize to the cold conditions, potentially exploiting the nutrients present in the rumen for replication and growth. The improvement in their adaptive abilities likely contributes to the observed increase in their relative abundance over the duration of storage.

The *Rikenellaceae RC9 gut group* is a disease-resistant microorganism in the gut, associated with lipid metabolism in the rumen of cattle, and its abundance correlates with feed efficiency [28,29]. These studies suggest that this group may enhance the microorganism’s adaptability to adverse environments, such as the freezing conditions in this study, by improving nutritional metabolism. Consequently, the relative abundance of this bacterium tends to increase overall. The abundance of the *Christensenellaceae R-7 group* correlates positively with feed-derived protein metabolism [30]. This group also decomposes undigested fiber in the intestines, thereby aiding the body in energy acquisition and improving nutrient utilization efficiency [31]. In the first 30 days of storage, there is a gradual decrease in the proteomic components of feed residues within the rumen fluid, characterized by reduced microbial protein content [23], which corresponds to a similar decline in the *Christensenellaceae R-7 group*’s abundance. After the 30-day period, as the fibrous components of feed residues in the rumen fluid are progressively broken down and utilized, the *Christensenellaceae R-7 group*, recognized for its role in fiber decomposition, shows an increase in abundance. *Veillonellaceae UCG-001*, a bacterium resident in the rumen known for its ability to degrade fiber, exhibits an increase in abundance that correlates with the improved apparent digestibility of neutral detergent fiber and acid detergent fiber [32]. In the context of this study, the relative abundance of *Veillonellaceae UCG-001* increased progressively over the storage period, a trend that may be associated with the residual feed components in the rumen fluid. Rumen microorganisms generally prioritize the fermentation of easily degradable substances like starch before addressing more resistant materials, such as fiber [33]. As the storage time extends, the proportion of remaining fibrous substances in the rumen fluid increases, which is mirrored by the increasing abundance of *Veillonellaceae UCG-001*.

The LEfSe analysis provides a detailed dissection of the differential microbial composition in rumen fluid across a spectrum of taxonomic levels under varying storage conditions, including temperature and time. Both *Succinivibrionaceae UCG-002* and *Mogibacterium* have been observed to correlate positively with nitrogen utilization efficiency and feed efficiency, with their populations tending to flourish under high-concentrate feeding regimes [34,35]. The present study revealed a heightened abundance of these two microbial taxa in fresh rumen fluid, a phenomenon attributed to the higher concentration of feed residues in the fluid, which is consistent with the widely accepted fact that ruminants on high-concentrate diets frequently demonstrate superior feed efficiency [36]. Lachnospiraceae, including its member *Lachnospiraceae UCG-004*, Bacteroidaceae with its member *Bacteroides*, and the order Eubacteriales, which encompasses Anaerofustaceae and *Anaerofustis*, are renowned for their robust carbohydrate-degrading abilities [37,38,39]. They can convert complex organic substances into volatile fatty acids, supplying energy to the host organism and demonstrating their formidable environmental adaptability. When fresh rumen fluid transitions from ambient temperature to cryopreservation, the metabolic activity of these microorganisms may be enhanced when stored after 7 days, allowing them to adjust to the new environment. This adaptation is evidenced by a significant increase in their population levels.

Gammaproteobacteria members, Succinivibrionaceae and *Succinivibrio*, are significant microbial communities abundant in the gut. They play a crucial role in the breakdown of carbohydrates and are positively correlated with feed efficiency [40]. Aeromonadales, known for thriving in both freshwater and marine environments, are potential pathogens in aquaculture and demonstrate remarkable adaptability to various environmental conditions [41]. This study found that these microbes maintain high abundance levels after 30 days of storage at −80 °C in rumen fluid, suggesting that the rumen microbiota are progressively adapting to changes in environmental conditions and nutritional availability. Similarly, Campilobacterota is equipped with an abundance of electron transport chain enzymes, and its chemotactic and energy taxis abilities allow it to seek out more advantageous conditions for growth [42]. *Pyramidobacter*, on the other hand, demonstrates metabolic flexibility, enabling it to adapt its metabolism when dietary polysaccharides are scarce by tapping into host-derived polysaccharides, which aids in its long-term persistence in the gut [43]. Existing research findings elucidate the increased prevalence of these microbes and their associated species after 30 days of storage at −20 °C. Furthermore, the higher concentration of volatile fatty acids in samples stored at −20 °C compared to those at −80 °C [23] underscores the microbes’ efficient energy utilization and conversion capabilities. The Ruminococcaceae family is renowned for its pivotal role in the degradation of fibers and the production of short-chain fatty acids (SCFAs). They are adept at breaking down a diverse range of polysaccharides and dietary fibers, making them the principal generators of SCFAs [44]. *Anaerovibrio* plays a role in lipid hydrolysis within the rumen of ruminants and is also likely involved in the metabolism of cellulose [45]. The Bacillaceae family boasts a range of metabolic pathways that encompass various biosynthetic and degradative processes, including those for amino acid, fatty acid, and carbon metabolism. These metabolic activities facilitate the conversion of nutrients and the cycling of energy in the rumen, which in turn enhances the adaptability and stability of the environment [46]. The microbes mentioned earlier exhibited a surge in abundance after 60 days of storage, indicating that the fibers in the rumen fluid feed residues began to degrade further, fats underwent a gradual breakdown, and amino acid metabolism intensified following two months of cryopreservation; all adaptations to an environment with limited nutrients. This explanation is substantiated by the observation that the energy metabolism pathway exhibited a high level of activity for a period of up to 60 days in storage.

Members of the Muribaculaceae family are adept at generating SCFAs by metabolizing both endogenous and exogenous polysaccharides, including dietary fiber [47]. They also engage in symbiotic interactions with beneficial bacteria such as *Bifidobacterium* and *Lactobacillus*. This study found an increase in the prevalence of Muribaculaceae after a 120-day storage period, a phenomenon that may be attributed to the predominance of fiber in the residual rumen fluid at that time. This observation is consistent with the relative reduction in total volatile fatty acid levels when compared to the 60-day mark [23]. Clostridia ferments carbohydrate-rich feed, converting low-quality protein sources and non-protein nitrogen compounds like urea into high-quality microbial protein [48]. Studies have revealed a positive correlation between the *Lachnospiraceae XPB1014 group* and the daily excretion of ammonia nitrogen (NH_3_-N) [49]. An increase in the population of *Clostridium* and the *Lachnospiraceae XPB1014 group* in rumen fluid after 240 days of storage suggests a scarcity of nitrogenous compounds at that time. The microbial community compensates by enhancing nitrogen utilization efficiency, as evidenced by the reduced NH_3_-N levels and increased microbial protein content. The genomes of *Lachnospiraceae UCG-008*, *Saccharofermentans*, and *Monoglobus* harbor numerous genes associated with the carbohydrate-active enzyme family, which play a role in the breakdown of complex carbohydrates such as polysaccharides, arabinoxylans, hemicelluloses, fructans, chitin, and pectin [50,51,52]. These enzymes bolster the microbes’ ability to survive under nutrient-poor conditions. The notable rise in the abundance of these specific microbes after 120 days of storage indicates a depletion of residual nutrients in the rumen fluid, prompting the microbes to upregulate their utilization of fibers and pectin to bolster their resilience against adversity.

In comparison with the microbial response patterns observed in rumen fluid under the high-grain feeding regime reported by Qiu et al. [17], microbial diversity in rumen fluid under the high-forage feeding regime did not show significant differences in response to storage temperatures (−80 °C and −20 °C). Similarly, most bacterial genera (with the exception of Proteobacteria) exhibited no differences in their response to these storage temperatures. However, microbial diversity and community composition showed significant differences in response to storage time. These differences are likely due to variations in the physicochemical properties of rumen fluid and the nutrients present in feed residues under different feeding regimes [10,53], which subsequently influence the ecological environment of rumen microbes.

## 5. Conclusions

In summary, preserving rumen fluid at −80 °C and −20 °C did not significantly alter bacterial alpha-diversity or community composition. However, storage time did affect bacterial richness and the relative abundances of specific species over the 240-day period. Consequently, it is recommended that rumen bacterial analysis be performed within two weeks of sample collection to maximize the representativeness and accuracy of the samples.

## Figures and Tables

**Table 1:** The effect of rumen fluid storage time and temperature on the alpha-diversity of rumen microbiota.

Item		Storage Time ^1^							SEM ^2^	*p*-Value ^3^		
		D0	D7	D14	D30	D60	D120	D240		Time	Temperature	Interaction
Chao1	−80 °C	673.47	460.86	852.46	678.30	480.62	1060.26	656.52				
	−20 °C	673.47	495.97	920.03	722.57	416.60	765.32	713.46	103.238	<0.001	0.694	0.402
	Average	673.47 ab	478.425 b	886.25 a	700.44 ab	448.61 b	912.79 a	684.99 ab				
	P0 value ^4^		0.486	0.381	1.000	0.316	0.084	1.000				
Observed species	−80 °C	656.33	456.00	808.67	660.67	473.33	967.33	641.50				
	−20 °C	656.33	489.00	860.67	671.33	416.33	741.50	685.67	92.234	<0.001	0.685	0.599
	Average	656.33 ab	472.50 b	834.67 a	666.00 ab	444.83 b	854.42 a	663.58 ab				
	P0 value		0.421	0.458	1.000	0.259	0.133	1.000				
PD whole tree	−80 °C	55.15	45.89	59.76	53.45	43.88	66.55	54.16				
	−20 °C	55.15	47.72	60.59	50.85	40.69	58.50	55.01	4.532	0.001	0.551	0.890
	Average	55.15 abc	46.81 bc	60.17 ab	52.15 abc	42.28 c	62.53 a	54.58 abc				
	P0 value		0.517	0.919	0.994	0.084	0.420	1.000				
Shannon index	−80 °C	7.18	7.27	7.37	7.41	7.22	7.45	7.26				
	−20 °C	7.18	7.26	7.60	7.42	7.40	7.48	7.51	0.382	0.974	0.632	0.999
	Average	7.18	7.27	7.49	7.42	7.31	7.46	7.39				
	P0 value		1.000	0.983	0.996	1.000	0.969	0.994				
Simpson index	−80 °C	0.971	0.982	0.973	0.977	0.979	0.973	0.977				
	−20 °C	0.971	0.981	0.982	0.981	0.985	0.978	0.982	0.010	0.878	0.434	0.999
	Average	0.971	0.982	0.978	0.979	0.982	0.975	0.980				
	P0 value		0.921	0.994	0.978	0.899	0.998	0.933				

Notes: ^1^ D0, D7, D14, D30, D60, D120, D240 indicate the fresh rumen fluid was stored for 0 days, 7 days, 14 days, 30 days, 60 days, 120 days, and 240 days, respectively. ^2^ SEM, standard error of the mean. ^3^ Interaction denotes interaction effect between preservation time and storage time. ^4^ The P0 value signifies a pairwise comparison between frozen rumen fluid at a specific preservation time and freshly collected rumen fluid (D0); in the same row, identical lowercase letters indicate no significant difference, while different lowercase letters indicate significant differences.

**Table 2:** The effect of rumen fluid storage time and temperature on the rumen bacterial community composition at the level of phylum.

Item		Storage Time ^1^							SEM ^2^	*p*-Value ^3^		
		D0	D7	D14	D30	D60	D120	D240		Time	Temperature	Interaction
Bacteroidota	−80 °C	69.11	62.28	75.71	72.60	72.73	70.21	69.04				
	−20 °C	69.11	69.24	73.97	75.80	61.85	64.04	59.59	4.228	0.137	0.265	0.352
	Average	69.11	65.76	74.84	74.20	67.29	67.13	64.31				
	P0 value ^4^		0.984	0.815	0.884	0.999	0.997	0.798				
Firmicutes	−80 °C	25.81	33.36	21.03	22.66	23.18	25.20	26.43				
	−20 °C	25.81	27.54	23.07	20.44	34.58	31.81	36.11	4.040	0.132	0.163	0.332
	Average	25.81	30.45	22.05	21.55	28.88	28.50	31.27				
	P0 value		0.906	0.964	0.935	0.987	0.981	0.638				
Proteobacteria	−80 °C	2.34	1.51	1.57	2.82	2.18	1.78	1.74				
	−20 °C	2.34	0.74	1.25	1.69	1.16	0.93	0.76	0.462	0.031	0.006	0.817
	Average	2.34	1.13	1.41	2.25	1.67	1.35	1.25				
	P0 value		0.132	0.400	1.000	0.758	0.133	0.072				
Verrucomicrobiota	−80 °C	0.70	0.62	0.15	0.35	0.22	0.76	0.48				
	−20 °C	0.70	0.53	0.13	0.44	0.27	0.71	0.32	0.210	0.038	0.814	0.997
	Average	0.70 a	0.58 ab	0.14 b	0.39 ab	0.24 ab	0.74 a	0.40 ab				
	P0 value		0.996	0.118	0.751	0.307	1.000	0.572				
Desulfobacterota	−80 °C	0.29	0.36	0.32	0.25	0.31	0.34	0.44				
	−20 °C	0.29	0.34	0.38	0.24	0.63	0.59	0.60	0.146	0.353	0.181	0.852
	Average	0.29	0.35	0.35	0.25	0.47	0.46	0.52				
	P0 value		0.999	1.000	1.000	0.872	0.751	0.445				
Spirochaetota	−80 °C	0.46	0.41	0.36	0.52	0.37	0.43	0.25				
	−20 °C	0.46	0.43	0.43	0.34	0.20	0.51	0.34	0.101	0.244	0.815	0.743
	Average	0.46	0.42	0.40	0.43	0.29	0.47	0.29				
	P0 value		0.999	0.994	1.000	0.589	1.000	0.390				
Synergistota	−80 °C	0.36	0.54	0.33	0.21	0.32	0.31	0.50				
	−20 °C	0.36	0.35	0.21	0.21	0.22	0.27	0.47	0.136	0.327	0.360	0.993
	Average	0.36	0.45	0.27	0.21	0.27	0.29	0.49				
	P0 value		0.996	0.991	0.910	0.990	0.994	0.925				
Actinobacteriota	−80 °C	0.17	0.18	0.15	0.05	0.17	0.25	0.33				
	−20 °C	0.17	0.25	0.16	0.25	0.52	0.43	0.82	0.219	0.279	0.127	0.860
	Average	0.17	0.22	0.16	0.15	0.34	0.34	0.58				
	P0 value		1.000	1.000	1.000	0.983	0.954	0.262				
Patescibacteria	−80 °C	0.11	0.16	0.08	0.14	0.16	0.18	0.30				
	−20 °C	0.11	0.15	0.11	0.16	0.26	0.25	0.50	0.065	<0.001	0.099	0.546
	Average	0.11 b	0.16 b	0.10 b	0.15 b	0.21 ab	0.21 ab	0.40 a				
	P0 value		0.991	1.000	0.996	0.712	0.430	<0.001				
Cyanobacteria	−80 °C	0.22	0.13	0.13	0.20	0.11	0.25	0.19				
	−20 °C	0.22	0.07	0.15	0.26	0.14	0.24	0.26	0.075	0.307	0.722	0.981
	Average	0.22	0.10	0.14	0.23	0.12	0.24	0.22				
	P0 value		0.624	0.924	1.000	0.818	1.000	1.000				

Notes: ^1^ D0, D7, D14, D30, D60, D120, D240 indicate the fresh rumen fluid was stored for 0 days, 7 days, 14 days, 30 days, 60 days, 120 days, and 240 days, respectively. ^2^ SEM, standard error of the mean. ^3^ Interaction denotes interaction effect between preservation time and storage time. ^4^ The P0 value signifies a pairwise comparison between frozen rumen fluid at a specific preservation time and freshly collected rumen fluid (D0); in the same row, identical lowercase letters indicate no significant difference, while different lowercase letters indicate significant differences.

**Table 3:** The effect of rumen fluid storage time and temperature on the rumen bacterial community composition at the level of genus.

Item		Storage Time ^1^							SEM ^2^	*p*-Value ^3^		
		D0	D7	D14	D30	D60	D120	D240		Time	Temperature	Interaction
*Prevotella*	−80 °C	43.03	34.87	54.33	45.54	48.22	41.86	39.60				
	−20 °C	43.03	40.71	51.76	46.16	34.59	36.61	30.05	7.7617	0.355	0.409	0.919
	Average	43.03	37.79	53.04	45.85	41.40	39.23	34.83				
	P0 value ^4^		0.993	0.847	1.000	1.000	0.996	0.845				
*Rikenellaceae RC9 gut group*	−80 °C	6.97	8.30	6.89	9.64	7.96	7.64	10.46				
	−20 °C	6.97	9.06	8.13	10.45	10.96	9.64	12.89	1.6991	0.048	0.119	0.968
	Average	6.97 b	8.68 ab	7.51 ab	10.04 ab	9.46 ab	8.64 ab	11.67 a				
	P0 value		0.948	1.000	0.539	0.757	0.885	0.021				
*Prevotellaceae UCG-003*	−80 °C	4.82	4.25	3.08	3.81	3.60	5.83	5.86				
	−20 °C	4.82	4.54	2.97	4.71	3.51	3.99	4.13	1.6956	0.895	0.690	0.970
	Average	4.82	4.39	3.02	4.26	3.55	4.91	4.99				
	P0 value		1.000	0.933	1.000	0.988	1.000	1.000				
*Christensenellaceae R-7 group*	−80 °C	6.58	8.27	1.59	1.58	1.65	3.52	3.36				
	−20 °C	6.58	7.06	1.67	0.71	5.16	4.75	5.82	1.1726	<0.001	0.250	0.430
	Average	6.58 ab	7.67 a	1.63 c	1.15 c	3.40 bc	4.14 abc	4.59 abc				
	P0 value		0.965	0.002	<0.001	0.112	0.157	0.369				
*Prevotellaceae UCG-001*	−80 °C	3.71	2.44	3.79	2.16	3.67	3.81	3.44				
	−20 °C	3.71	2.06	3.12	2.87	2.07	2.55	2.06	1.6449	0.973	0.467	0.992
	Average	3.71	2.25	3.45	2.51	2.87	3.18	2.75				
	P0 value		0.972	1.000	0.990	0.998	1.000	0.991				
*Succiniclasticum*	−80 °C	1.83	2.80	2.92	1.69	2.72	2.86	3.15				
	−20 °C	1.83	1.82	2.63	2.47	2.32	2.63	2.95	0.7633	0.552	0.652	0.979
	Average	1.83	2.31	2.77	2.08	2.52	2.75	3.05				
	P0 value		0.996	0.873	1.000	0.969	0.754	0.450				
*Selenomonas*	−80 °C	1.35	1.80	2.48	3.84	3.38	2.60	2.17				
	−20 °C	1.35	1.33	2.70	1.69	2.13	1.90	1.48	0.8247	0.432	0.114	0.876
	Average	1.35	1.57	2.59	2.77	2.76	2.25	1.83				
	P0 value		1.000	0.735	0.600	0.608	0.827	0.991				
*NK4A214 group*	−80 °C	2.51	2.97	1.28	1.36	1.26	1.21	1.90				
	−20 °C	2.51	2.49	1.13	1.23	2.46	2.17	3.10	0.8231	0.390	0.411	0.881
	Average	2.51	2.73	1.21	1.29	1.86	1.69	2.50				
	P0 value		1.000	0.685	0.749	0.985	0.880	1.000				
*Veillonellaceae UCG-001*	−80 °C	0.98	1.30	1.82	1.34	1.74	1.96	2.15				
	−20 °C	0.98	0.92	1.70	1.80	1.81	1.87	2.65	0.4267	0.006	0.792	0.942
	Average	0.98 b	1.11 ab	1.76 ab	1.57 ab	1.77 ab	1.91 ab	2.40 a				
	P0 value		1.000	0.525	0.796	0.497	0.118	0.003				

Notes: ^1^ D0, D7, D14, D30, D60, D120, D240 indicate the fresh rumen fluid was stored for 0 days, 7 days, 14 days, 30 days, 60 days, 120 days, and 240 days, respectively. ^2^ SEM, standard error of the mean. ^3^ Interaction denotes interaction effect between preservation time and storage time. ^4^ The P0 value signifies a pairwise comparison between frozen rumen fluid at a specific preservation time and freshly collected rumen fluid (D0); in the same row, identical lowercase letters indicate no significant difference, while different lowercase letters indicate significant differences.

**Table 4:** The effect of rumen fluid storage time and temperature on the relative abundance of the predicted metabolic pathways in the rumen bacterial microbiome.

Item		Storage Time ^1^							SEM ^2^	*p*-Value ^3^		
		D0	D7	D14	D30	D60	D120	D240		Time	Temperature	Interaction
Metabolism of cofactors and vitamins	−80 °C	14.87	14.70	15.07	14.87	14.96	14.80	14.77				
	−20 °C	14.87	14.81	14.97	15.02	14.33	14.44	14.12	0.239	0.160	0.111	0.427
	Average	14.87	14.75	15.02	14.95	14.64	14.62	14.44				
	P0 value ^4^		0.999	0.996	1.000	0.958	0.851	0.313				
Carbohydrate metabolism	−80 °C	14.03	13.82	14.07	14.01	14.09	13.83	13.90				
	−20 °C	14.03	13.93	14.11	14.08	14.05	13.96	13.83	0.152	0.530	0.678	0.985
	Average	14.03	13.87	14.09	14.05	14.07	13.89	13.87				
	P0 value		0.946	0.999	1.000	1.000	0.935	0.854				
Amino acid metabolism	−80 °C	13.05	13.01	13.03	13.03	13.08	12.92	13.02				
	−20 °C	13.05	13.00	13.07	13.08	13.10	13.03	13.00	0.110	0.954	0.637	0.992
	Average	13.05	13.01	13.05	13.06	13.09	12.98	13.01				
	P0 value		1.000	1.000	1.000	1.000	0.981	1.000				
Metabolism of terpenoids and polyketides	−80 °C	8.64	8.83	8.15	8.49	8.34	8.70	8.53				
	−20 °C	8.64	8.63	8.27	8.42	8.77	8.74	9.03	0.217	0.176	0.324	0.610
	Average	8.64	8.73	8.21	8.45	8.55	8.72	8.78				
	P0 value		0.999	0.425	0.977	1.000	0.999	0.980				
Metabolism of other amino acids	−80 °C	6.72	6.75	6.68	6.72	6.94	6.86	6.83				
	−20 °C	6.72	6.69	6.71	6.71	6.73	6.86	7.04	0.118	0.191	0.941	0.723
	Average	6.72	6.72	6.70	6.71	6.84	6.86	6.94				
	P0 value		1.000	1.000	1.000	0.958	0.775	0.285				
Replication and repair	−80 °C	6.51	6.54	6.47	6.47	6.47	6.42	6.45				
	−20 °C	6.51	6.59	6.50	6.52	6.47	6.46	6.41	0.052	0.145	0.532	0.949
	Average	6.51	6.57	6.49	6.50	6.47	6.44	6.43				
	P0 value		0.888	1.000	1.000	0.994	0.652	0.562				
Glycan biosynthesis and metabolism	−80 °C	6.21	5.98	6.46	6.25	6.33	6.18	6.08				
	−20 °C	6.21	6.19	6.37	6.38	5.70	5.82	5.52	0.250	0.195	0.175	0.521
	Average	6.21	6.08	6.41	6.31	6.01	6.00	5.80				
	P0 value		0.999	0.980	0.999	0.987	0.948	0.425				
Energy metabolism	−80 °C	5.69	5.69	6.05	6.04	6.06	6.01	6.04				
	−20 °C	5.69	5.94	6.06	5.71	6.01	5.99	5.94	0.099	0.001	0.531	0.329
	Average	5.69 b	5.81 ab	6.05 a	5.88 ab	6.03 a	6.00 a	5.99 ab				
	P0 value		0.853	0.009	0.464	0.014	0.005	0.008				
Lipid metabolism	−80 °C	4.03	4.28	3.45	3.99	3.59	4.05	3.88				
	−20 °C	4.03	4.08	3.84	3.97	4.24	4.06	4.30	0.209	0.382	0.120	0.434
	Average	4.03	4.18	3.65	3.98	3.92	4.05	4.09				
	P0 value		0.990	0.525	1.000	0.998	1.000	1.000				
Translation	−80 °C	3.52	3.58	3.48	3.49	3.50	3.48	3.51				
	−20 °C	3.52	3.57	3.49	3.52	3.52	3.51	3.50	0.022	0.011	0.337	0.957
	Average	3.52 ab	3.58 a	3.49 b	3.50 b	3.51 ab	3.49 b	3.50 b				
	P0 value		0.217	0.613	0.970	0.991	0.629	0.885				

Notes: ^1^ D0, D7, D14, D30, D60, D120, D240 indicate the fresh rumen fluid was stored for 0 days, 7 days, 14 days, 30 days, 60 days, 120 days, and 240 days, respectively. ^2^ SEM, standard error of the mean. ^3^ Interaction denotes interaction effect between preservation time and storage time. ^4^ The P0 value signifies a pairwise comparison between frozen rumen fluid at a specific preservation time and freshly collected rumen fluid (D0); in the same row, identical lowercase letters indicate no significant difference, while different lowercase letters indicate significant differences.

## Data Availability

The raw sequencing data have been deposited in the NCBI’s Sequence Read Archive (SRA) database under accession number PRJNA982413.

## Abbreviations

The following abbreviations are used in this manuscript:

NH_3_-N	Ammonia nitrogen
ASVs	Amplicon sequence variants
ANOSIM	Analysis of similarities
NMDS	Non-metric multidimensional scaling
PD	Phylogenetic diversity
PCoA	Principal coordinates analysis
rRNA	ribosomal RNA
SCFAs	Short-chain fatty acids
